# Supplementary material for: Differential Immune Checkpoint and Ig-like V-Type Receptor Profiles in COVID-19: Associations with Severity and Treatment
Source: J Clin Med. 2022 Jun 8;11(12):3287. doi: 10.3390/jcm11123287 (PMC9225268; doi:10.3390/jcm11123287)
Supplement: Supplementary file 1 [file jcm-11-03287-s001.zip › Supplementary table S4 and S5 Clinical parameters and soluble markers associated to severity.pdf]

Supplementary Table S4. Clinical baseline characteristics and routine laboratory data at admission in COVID-19 patients classified according to their severity and final outcome.

Type		No hospitalized (n=22)	Hospitalized with no O_2 (n=10)	O_2 requirement (n=23)	Orotracheal intubation (n=13)	*Exitus* (n=19)	P-value
Baseline characteristics	Age (years)	56 (49-72)	51 (44-66)	69 (55-77)	60 (56-69)	73 (63-87)	0.01#
	qSOFA > 1	0 (0.0%)	1 (10%)	3 (13%)	1 (7.7%)	7 (36.8%)	0.01$
	$SatO_2$ (%)	97 (94-98)	96 (94-98)	95 (93-96)	94 (85-96)	91 (89-94)	< 0.01*
	SpO_2/FiO_2	462 (446-467)	450 (441-466)	447.6 (441-457)	328.6 (307-405)	342.9 (242-431)	**0.02***
	Resp. Rate	20 (18-22)	20 (18-25)	22 (20-26)	22 (18-28)	25 (22-28)	< 0.01*
Inflammation and other markers	PCT	0.02 (0.02-0.05)	0.15 (0.03-0.45)	0.06 (0.05-0.36)	0.32 (0.28-0.8)	0.27 (0.15-2.32)	**< 0.01***
	CRP	5.1 (1.2-23.4)	45 (26-105)	29.3 (12-94)	179.7 (52-234)	117.3 (46-167)	**< 0.01***
	Ferritin	136 (63-281)	342 (177-374.3)	250.0 (105-507)	974 (574-2197)	776.0 (426-1373)	**< 0.01***
	Lactate	1.0 (1-1.1)	1.0 (0.9-1.1)	1.0 (0.91.2)	1 (0.7-1.5)	1.5 (1-2.6)	0.01*
	LDH	232 (199-292)	236 (207-327)	345 (286-377)	408 (280-444)	371 (287-545)	< 0.01*
	D-Dimer	390 (235-720)	489 (338-955)	750 (550-1285)	1290 (940-3840)	1850 (645-5395)	< 0.01*
	Platelets	288000 (222750-362500)	229000 (194000-339500)	242000 (160800-291500)	295000 (242000-381000)	185000 (110500-279500)	0.04#
	AST	23 (13.5-29)	29 (22.3-38)	41 (29.5-53)	40 (29-66)	33 (25.5-55)	0.01*
	Hemoglobin	14.2 (13.4-14.7)	13.3 (12.7-14.5)	14.1 (12.6-15)	12.7 (8.6-14.5)	12.8 (9.9-13.6)	0.01#
	RBC (%)	43.1 (40-45)	42.1 (39-44)	42.0 (37.8-45)	39.8 (27.5-44)	38.8 (30.8-42)	0.01#
Leukocytes	Lympho (%)	23.9 (17-28)	17.3 (10-23)	15.9 (11.1-20.4)	9.2 (5.9-14.8)	5.7 (4.9-12.4)	< 0.01#
	ALC	1385 (1045-1775)	830 (620-1102)	930 (655-1095)	720 (270-930)	420 (335-780)	< 0.01#
	Mono (%)	5.7 (4.9-7.5)	4.2 (2.8-6.8)	6.0 (4.3-7.7)	4.8 (3.5-6.1)	4.0 (2.8-5.3)	0.05#
	Neut (%)	64.8 (60-75)	71.7 (63-85)	74.4 (69.8-79)	78.6 (72.5-81.9)	84.9 (77.5-89.3)	< 0.01*
	ANC	4130 (3048-5960)	3435 (3038-5978)	3670 (3135-4715)	6450 (4240-6680)	6950 (4245-8520)	0.048*
	Eos (%)	0.8 (0.5-1.6)	0.2 (0.1-0.7)	0.2 (0.2-0.7)	0.6 (0.2-5.1)	0.3 (0.1-0.8)	0.01*
	AEC	50 (30-100)	15 (1.5-55)	20 (10-50)	50 (10-180)	10 (0.5-60)	0.03*
	NLR	2.8 (2.2-4.4)	4.4 (2.2-8.6)	4.3 (3.3-7.2)	8.92 (6.6-15.2)	15.4 (8.8-18.6)	**< 0.01***

Data as Median (IQR) or number (Percentage). * in Kruskal-Wallis test; # in One-way ANOVA and $ in Xi2 + Cramer's V. In bold, those p-values with high significance (defined by p-value<FDR by Benjamini-Hochberg method. qSOFA, quick sequential organ failure assessment score; $SatO_2$, oxygen saturation; SpO_2/FiO_2, peripheral blood oxygen saturation to fraction of inspired oxygen ratio; PCT, Procalcitonin; CRP, C-reactive protein; LDH, lactate dehydrogenase; AST, aspartate transaminase; RBC, hematocrit; ALC, absolute lymphocyte count; AMC, absolute monocyte count; ANC, absolute neutrophil count; AEC, absolute eosinophil count; NLR, neutrophil-lymphocyte ratio. Units: PCT, Ferritin and D-Dimer in ng/mL; CRP in mg/L, Lactate in nmol/L, LDH and AST in U/L; Platelets, ALC, ANC and AEC in counts/mm^3; Hemoglobin in g/dL.

Supplementary Table S5. Plasma soluble markers at admission in COVID-19 patients classified according to their severity and final outcome.

Type		No hospitalized (n=22)	Hospitalized with no O_2 (n=10)	O_2 requirement (n=23)	Orotracheal intubation (n=13)	*Exitus* (n=19)	P-value
Chemokines and cytokines	CCL2	130.5 (100-156)	85.2 (62-107)	121.9 (58-230)	189.5 (146-333)	322.4 (130-775)	< 0.01*
	CXCL10	84.7 (52-165)	254.6 (102-416)	229.3 (88-689)	154.9 (109-657)	386.1 (165-795)	0.01*
	IL-2	4 (0-5.3)	0.0 (0-0)	0.0 (0-2.9)	6.3 (4-17)	5.1 (0-14)	< 0.01*
	IL-6	59.5 (0-77)	5.6 (0.8-15)	38.6 (6.1-83)	92.4 (63-261)	146.3 (46-550)	< 0.01*
	IL-8	11.3 (0-22)	0.0 (0-1.8)	8.2 (0-19)	24.9 (24-120)	44.8 (3-181)	< 0.01*
	IL-10	9.7 (0-11.6)	0.5 (0-2.6)	5.2 (2-10.3)	8.5 (7.2-15)	11.6 (1-16)	0.01*
	IL-12p70	11.9 (0-17.9)	0.0 (0-0)	0.0 (0-8)	8.5 (0-12)	0.0 (0-12)	0.02*
Immune *checkpoints*	sCD25	981.3 (654-1422)	1001.3 (888-1203)	1095.1 (651-1639)	1752 (936-2290)	2609 (1417-3177)	< 0.01*
	sTim-3	5904 (3075-7478)	10611 (5642-17528)	11347 (5727-15374)	14916 (9115-21705)	20646 (11925-43314)	< 0.01*
	Gal-9	37694 (23015-45969)	91315 (30258-135332)	76326 (54214-170860)	90527 (48718-159358)	119095 (62468-186572)	< 0.01*
Thrombosis	tPA	3209 (2057-4731)	6305 (3044-9890)	9225 (2633-17484)	8321 (4078-23899)	9513 (4667-33427)	0.01*
	P-Selectin	6157 (562-11898)	979 (0-11384)	22679 (3782-37749)	17581 (8958-21027)	14401 (2206-29236)	0.03*

Data are pg/mL expressed as Median (IQR). IL, interleukin; Gal-9, Galectin-9; tPA, tissue plasminogen activator; * Kruskal-Wallis test.
