# Supplementary material for: Differential Immune Checkpoint and Ig-like V-Type Receptor Profiles in COVID-19: Associations with Severity and Treatment
Source: J Clin Med. 2022 Jun 8;11(12):3287. doi: 10.3390/jcm11123287 (PMC9225268; doi:10.3390/jcm11123287)
Supplement: Supplementary file 1 [file jcm-11-03287-s001.zip › Supplementary table S6 and S7 Clinical paramerts and soluble markers associated to secondary infections.pdf]

Supplementary Table S6. Clinical baseline characteristics and routine laboratory data at admission in COVID-19 patients classified according to whether they developed a secondary infection during their hospital stay.

Type		No secondary infection (n=54)	Secondary infection (n=33)	P-value
Baseline characteristics	Sex (male)	19 (35.8)	21 (63.3)	0.02$
	GCS (<14)	4 (7.4%)	10 (30.3%)	0.01$
	SOFA score (=0)	36 (66.7%)	8 (33.3%)	< 0.01$
	SpO2/FiO2	454.8 (434-462)	384 (257-443)	**< 0.01***
	Heart rate	89 (77.5-102)	105 (90-110)	< 0.01#
	SBP	126.5 (114-146)	116 (102-126)	0.01#
Inflammation and other markers	PCT	0.05 (0.02-0.3)	0.28 (0.07-0.66)	< 0.01*
	CRP	33.25 (6.4-93.7)	86.7 (24.7-208)	0.01*
	Ferritin	251 (121-403)	580 (250-1408)	< 0.01*
	LDH	281.5 (214-352)	338 (280-520)	0.01*
	pH	7.44 (7.42-7.5)	7.41 (7.31-7.45)	0.02*
	D-Dimer	600 (366-1108)	1200 (610-5030)	< 0.01*
	Hemoglobin	13.95 (12.8-14.95)	12.8 (9.1-14.1)	< 0.01#
	RBC (%)	42.1 (39-45)	39.2 (29.7-43.5)	< 0.01#
Leukocytes	ALeC	5700 (4208-7680)	7500 (4950-9680)	0.02*
	Lympho (%)	18.55 (13.9-25.8)	8.9 (5.2-13.7)	**< 0.01#**
	Mono (%)	5.65 (4.4-7.38)	4.3 (2.8-5.8)	0.01#
	Neut (%)	71.55 (63-78)	80.9 (74.5-88)	< 0.01*
	ANC	4020 (3013-5960)	6450 (3630-7880)	< 0.01*
	NLR	4.02 (2.35-6.4)	8.95 (5.3-15.4)	< 0.01*

Data as Median (IQR) or number (Percentage), * in Mann-Whitney test; # in One-way ANOVA and $ in Xi^2 + Cramer's V. In bold, those p-values with high significance (defined by p-value<FDR by Benjamini-Hochberg method). GCS, Glasgow coma score; SpO_2/FiO_2, peripheral blood oxygen saturation to fraction of inspired oxygen ratio; HR, Heart Rate; SBP, Systolic blood pressure; PCT, Procalcitonin; CRP, C-reactive protein; LDH, lactate dehydrogenase; RBC, hematocrit; ALeC, absolute leukocyte count; ANC, absolute neutrophil count; NLR, Neutrophil-Lymphocyte Ratio. Units: PCT, Ferritin and D-Dimer in ng/mL; CRP in mg/L, LDH and; Platelets, ALeC and ANC in counts/mm3; Hemoglobin in g/dL; SBP in mmHg; HR in beats per min.

Supplementary Table S7. Plasma soluble markers at admission in COVID-19 patients classified according to whether they developed a secondary infection during their hospital stay.

Type		No secondary infection (n=54)	Secondary infection (n=33)	P-value
Chemokines and cytokines	CCL2	121.9 (82.8-186)	194.5 (145-364)	< 0.01*
	IFNγ	5.27 (0-36.3)	36.05 (19.9-48)	< 0.01
	IL-1β	0 (0-0.4)	0 (0-6.88)	0.02*
	IL-2	0 (0-4.35)	5.3 (1.07-17.2)	< 0.01*
	IL-4	2.75 (0-17)	19.7 (2.1-30)	0.03*
	IL-6	23.6 (3.6-78)	93.4 (56.6-242.7)	< 0.01*
	IL-8	5.8 (0-16)	34.8 (10.1-139)	< 0.01*
	IL-10	0 (0-9.25)	8.54 (0-13.38)	0.02*
	IL-12p70	3.13 (0-10)	11.06 (6.46-15)	< 0.01*
	IL-17a	0.82 (0-6.7)	5.83 (1.43-8.7)	0.01*
Immune checkpoint	sTim-3	8559 (4915-16147)	14802 (9115-23863)	0.03*

Data are pg/mL expressed as Median (IQR). IFN, interferon; IL, interleukin; * Mann-Whitney test.
