# Supplementary material for: Differential Immune Checkpoint and Ig-like V-Type Receptor Profiles in COVID-19: Associations with Severity and Treatment
Source: J Clin Med. 2022 Jun 8;11(12):3287. doi: 10.3390/jcm11123287 (PMC9225268; doi:10.3390/jcm11123287)
Supplement: Supplementary file 1 [file jcm-11-03287-s001.zip › Supplementary tables S2 and S3.pdf]

Supplementary Table S2. List of all fluorochrome-conjugated monoclonal antibodies for flow cytometry analysis of immune subpopulation.

**Marker**	**Fluorochrome**	**Source**	**Clone**	**Catalog number**
CD45RA	BUV395	BD	5H9	740315
CD16	BUV496	BD	3G8	612944
CD195 (CCR5)	BUV563	BD	2D7/CCR5	741401
CD62L	BV615	BD	SK11	751364
CD11c	BUV661	BD	B-ly6	612967
CD56	BUV737	BD	NCAM16.2	612766
CD8	BUV805	BD	SK1	612889
IgD	BV480	BD	IA6-2	566138
IgG	BV7605	BD	G18-145	563246
CD185 (CXCR5)	BV750	BD	RF8B2	747111
CD141	BB515	BD	1A4	566017
CD127	APC-R700	BD	HIL-7R-M21	565185
CD197 (CCR7)	BV421	Biolegend	G043H7	353208
CD3	BV510	Biolegend	OKT3	317332
IgM	BV570	Biolegend	MHM-88	314517
CD28	BV650	Biolegend	CD28.2	302946
CD196 (CCR6)	BV711	Biolegend	G034E3	353436
CD279 (PD-1)	BV785	Biolegend	EH12.2H7	329929
CD57	FITC	Biolegend	HNK-1	359604
CD14	SparkBlue550	Biolegend	63D3	367148
CD45	PerCP	Biolegend	2D1	368506
CD11b	PCPCy5.5	Biolegend	ICRF44	301328
CD274 (PD-L1)	PE	Biolegend	10F.9G2	124308
CD24	PEDz594	Biolegend	ML5	311134
CD95 (Fas)	PECy5	Biolegend	DX2	305610
CD183 (CXCR3)	PECy7	Biolegend	G025H7	353720
CD27	APC	Biolegend	M-T271	356410
CD1c	AF647	Biolegend	L161	331510
CD19	SparkNIR685	Biolegend	HIB19	302270
HLA-DR	APCF750	Biolegend	L243	307658
CD38	APC-Fire810	Biolegend	L243	356644
CD4	CF568	Cytek	SK3	SKU R7-20042
CD123	Super Bright 436	ThermoFisher	6H6	62-1239-42
CD161	eFluor450	ThermoFisher	HP-3G10	48-1619-42
CD20	Pacific Orange	ThermoFisher	HI47	MHCD2030
TCRgd	PCPCeF710	ThermoFisher	B1.1	46-9959-42
CD25	PE-AF700	ThermoFisher	CD25-3G10	MHCD2524

AF, AlexaFluor; BV, Brilliant Violet; BUV, Brilliant Ultra Violet; SB, super bright

Supplementary Table S3. List of all fluorochrome-conjugated monoclonal antibodies for flow cytometry analysis of immune-checkpoints and other IgG like V type markers expression on immune populations.

Marker	Fluorochrome	Source	Clone	Catalog number
CD162 (PSGL-1)	BV395	BD	KPL-1	743484
HLA-DR	BV496	BD	G46-6	749866
CD276 (B7-H3)	BUV563	BD	7-517	748380
CD134	BUV661	BD	ACT35	750645
VISTA (B7-H5)	BUV737	BD	MIH65.rMAb	749648
CD108 (SEMA7A)	BUV805	BD	KS-2	749451
TIGIT	BV421	Biolegend	A15153G	372710
CD1c	SB436	ThermoFisher	L161	62-0015-42
CD270 (HVEM)	BV480	BD	CW10	746607
CD16	BV510	Biolegend	3G8	302048
CD8	Pacific Orange	ThermoFisher	3B5	MHCD0830
CD4	BV570	Biolegend	RPA-T4	300534
CD33 (Siglec-3)	BV605	Biolegend	P67.6	366612
CD321 (JAM-1)	BV650	BD	M.Ab.F11	744066
CD206 (MMR)	BV711	Biolegend	15-2	321136
CD56	BV750	Biolegend	5.1H11	362556
CD137	BV786	BD	4B4-1	741000
CD14	AF488	Biolegend	63D3	367130
CD3	SparkBlue550	Biolegend	SK7	344852
CD11b	PerCp	Biolegend	M1/70	101230
CD155	PerCp-eFluor710	ThermoFisher	2H7CD155	46-1550-42
GITR-L (TNFSF18)	PE	R&D Systems	109101	FAB6941P
CD226 (DNAM-1)	PE/Dazzle™ 594	Biolegend	11A8	338318
CD357 (GITR)	PE/Cyanine5	Biolegend	621	311608
CD158b (KIR2DL2)	PE/Cyanine7	Biolegend	DX27	312610
CD170 (Siglec-5)	APC	Biolegend	1A5	352006
CD152 (CTLA-4)	AF647	Biolegend	BNI3	369626
LAG-3 (CD223)	APC-R700	BD	T47-530	565774
CD366 (Tim-3)	APC-eFluor780	ThermoFisher	F38-2E2	47-3109-42

AF, AlexaFluor; BV, Brilliant Violet; BUV, Brilliant Ultra Violet; SB, super bright
